# Electroacupuncture Alleviates Depressive-Like Symptoms and Modulates BDNF Signaling in 6-Hydroxydopamine Rats

**DOI:** 10.1155/2016/7842362

**Published:** 2016-07-25

**Authors:** Min Sun, Ke Wang, Yan Yu, Wen-Ting Su, Xin-Xin Jiang, Jian Yang, Jun Jia, Xiao-Min Wang

**Affiliations:** ^1^Departments of Neurobiology and Physiology, Key Laboratory for Neurodegenerative Disorders of the Ministry of Education, Beijing Key Laboratory for Parkinson's Disease, Beijing Institute for Brain Disorders, Capital Medical University, Beijing 100069, China; ^2^Beijing Key Laboratory for Mental Disorders, China Clinical Research Center for Mental Disorders, Beijing Anding Hospital, Capital Medical University, 5 Ankang Alley, Beijing 100088, China

## Abstract

Previous studies have identified the beneficial effects of electroacupuncture (EA) on motor behaviors in Parkinson's disease (PD). However, the role and potential mechanisms of EA in PD-associated depression remain unclear. In the present study, a rat model of PD with unilateral 6-hydroxydopamine (6-OHDA) lesions in the medial forebrain bundle was treated using EA for 4 weeks. We found that 100 Hz EA improved several motor phenotypes. In addition, tyrosine hydroxylase (TH) immunohistochemical analysis showed that EA had a minimal impact on the TH-positive profiles of the ipsilateral ventral tegmental area. Compared with the 6-OHDA group, long-term EA stimulation significantly increased sucrose solution consumption and decreased immobility time in the forced swim test. EA treatment did not alter dopamine, norepinephrine, and serotonin levels in the striatum and hippocampus. Noticeably, EA treatment reversed the 6-OHDA-induced abnormal expression of brain-derived neurotrophic factor (BDNF) and tropomyosin-related kinase B (TrkB) in the midbrain and hippocampus. These results demonstrate that EA at 100-Hz possesses the ability to improve depressive-like symptoms in PD rats, which is, at least in part, due to the distinct effect of EA on the mesostriatal and mesocorticolimbic dopaminergic pathways. Moreover, BDNF seems to participate in the effect of EA in PD.

## 1. Introduction 

Parkinson's disease (PD) is generally recognized as a progressive neurodegenerative disorder characterized by motor dysfunction. However, accumulating evidence indicates that nonmotor symptoms of PD, such as sensory dysfunction, sleep disturbance, and psychiatric complications (depression, anxiety, apathy, and cognitive impairment), are more detrimental to well-being and functional ability than motor symptoms [1–3]. As one of the most common comorbidities, depression affects approximately 40–50% of PD patients [4] and might account for substantial disability and poor quality of life. Currently, selective serotonin reuptake inhibitors and tricyclic antidepressants are the two main categories of antidepressants used for treating depression in PD. However, the efficacy of antidepressant treatment in PD remains unclear [5]. Besides, polypharmacy, with antidepressant and antiparkinsonian treatments, may produce unexpected drug interactions and complicate disease development [6].

Acupuncture, a traditional complementary and alternative medical approach, has been widely used as an adjunct to standard therapy for PD. Although the definite role of acupuncture in PD is controversial [7], increasing evidence shows that acupuncture or electroacupuncture (EA) can alleviate motor and nonmotor symptoms, including tremors, slowness, pain, depression, sleep disturbance [8], and anxiety [9], and is particularly effective at early stages in PD patients [10]. Prior experiments have indicated that high-frequency EA stimulation (100 Hz) is effective in improving motor function in various animal models of PD by normalizing neurotransmitters in the basal ganglia [11], inhibiting neuroinflammatory responses [12], reducing oxidative stress [13, 14], and increasing neurotrophic factors [15], among other methods of action. However, few studies have investigated the exact effects of EA on nonmotor symptoms, particularly depressive symptoms.

Brain-derived neurotrophic factor (BDNF), a member of the neurotrophin family, plays critical roles in cell differentiation, neuronal survival, migration, and synaptic plasticity. BDNF is reportedly involved in the pathophysiology of both PD and depression, as evidenced by the fact that altered expression of BDNF mRNA and protein has been observed in postmortem studies of PD patients [16]. Meanwhile, reduced expression of BDNF mRNA and protein has been found in the mesostriatal and mesocorticolimbic pathways, as well as in the serum of patients with depression [17, 18]. Further alterations in BDNF expression have been observed in the mesocorticolimbic and nigrostriatal systems in experimental animals [19–22]. BDNF needs to bind to its high-affinity protein kinase receptor, tropomyosin-related kinase B (TrkB), to exert its biological effects [23]. Although the precise mechanism is under investigation, abnormalities in BDNF-TrkB signaling may substantially contribute to the development of depression in PD.

Animal models are an important way to improve our understanding of the pathophysiological mechanisms of PD and possible treatments. Studies have suggested that bilateral infusion of 6-hydroxydopamine (6-OHDA) into the striatum [24], SNc [25, 26], or ventral tegmental area (VTA) [27, 28] produces a depressive-like phenotype in rats. Among PD models, the rat model of a unilateral 6-OHDA lesion in the medial forebrain bundle (MFB) has lesions in both the mesostriatal and mesocorticolimbic dopaminergic pathways [29, 30]. This lesion protocol is known to cause significant motor features and several nonmotor impairments of PD, including depression, apathy, and anhedonia [31]. In the present study, we evaluated the effect of EA treatment on both motor and nonmotor behaviors in the unilateral MFB-lesioned PD rat model. We have also investigated the changes of these neurotransmitters including dopamine (DA), norepinephrine (NE), and serotonin (5-HT) in the striatum and hippocampus after 4 weeks of EA treatment. In addition, the effects of EA treatment on BDNF-TrkB signals within the midbrain and hippocampus were examined. The purpose of this study is to explore the effects of EA treatment on the motor and nonmotor phenotypes and elucidate the potential antidepressive mechanism mediated by EA in PD.

## 2. Methods

### 2.1. Animals and Unilateral 6-OHDA Lesion Induction

Adult Sprague-Dawley (SD) rats weighing 200–220 g were obtained from the animal facility of Capital Medical University. Rats were housed three per cage under standard laboratory conditions: 12-h light/dark cycle, 22°C room temperature, 55% relative humidity, and access to food and water* ad libitum*. All experimental procedures were performed according to the Ethics Committee on Animal Care and Usage of Capital Medical University.

Rats were anesthetized with 2% pentobarbital sodium (40 mg/kg, i.p.) and fixed into a Kopf stereotaxic apparatus. Lesions were induced by injection of 6-OHDA using a 30-gauge needle Hamilton syringe into the right MFB at the following coordinates: anteroposterior (AP), –4.3 mm; mediolateral (ML), –1.5 mm; and dorsoventral (DV), 7.6 mm from the dura. A total dose of 8 *μ*g (5 *μ*g/*μ*L, 1.6 *μ*L) 6-OHDA (Sigma-Aldrich) was administered, at a rate of 0.5 *μ*L/min. The microsyringe was left in place for 4 min to allow diffusion. Sham-operated rats underwent the same surgical procedure but received saline injection. Behaviors were assessed 2 weeks after surgery.

### 2.2. EA Stimulation

EA stimulation was administered from 2 weeks to 6 weeks after 6-OHDA lesions were induced in the rats. Rats were randomly divided into 4 groups: a vehicle-treated sham group, a 6-OHDA-lesioned model group, and two groups of 6-OHDA-lesioned rats receiving EA at either 0 or 100 Hz. Two stainless steel needles of diameter 0.25 mm were inserted at a depth of 5 mm into the acupoints of BAIHUI (GV 20, at the midpoint between the auricular apices) and DAZHUI (GV 14, directly below the spinous process of the vertebra prominens). A bidirectional square wave (0.2 ms) electrical pulse with frequency 100 Hz was administered from a medical EA apparatus (HANS, Neuroscience Research Institute, Beijing, China) for 30 min a day, 6 days a week for 4 weeks. The intensity of stimulation was increased stepwise from 1 to 2 mA and then to 3 mA, with each step lasting for 10 min. During EA stimulation, the rats were kept in the cage in an awake, unrestrained condition. Those treated with EA at 0 Hz underwent the same procedures, but no electrical pulses were delivered through the needles.

### 2.3. Behavioral Assessment

#### 2.3.1. Rotarod Test

The rotarod test was used to evaluate balance and motor coordination. The initial 3 days of testing served as training, and rats were placed on the rod turning at a low speed, permitting them to attain stable performance. On the test day, an accelerating protocol going from 4 rpm to 40 rpm in 2 min was performed, and the time taken for the rats to fall was automatically recorded.

#### 2.3.2. Open Field Test

To assess general locomotor activity and anxiety-related activity, the rats were monitored using automatic infrared beams in a black chamber (TruScan 2.0 Instruments, Columbus, OH). The rats were placed in the arena and allowed to freely move about for 30 min while being recorded by an overhead camera. The footage was then analyzed using DigiScan analyzer and software (TruScan 2.0, Columbus) to extract the following parameters: horizontal movement distance, vertical movement distance, and numbers of entries into the arena center.

#### 2.3.3. Apomorphine-Induced Turning Behavior

This behavioral test was performed in a blinded fashion. The rats received a subcutaneous injection of 0.05 mg/kg apomorphine hydrochloride (Sigma) dissolved in 1% ascorbic acid and 0.9% NaCl. Immediately after apomorphine injection, the net number of rotations was recorded and assessed over 30 min. Rotational testing was performed in automatic rotameter bowls (Coulbourn Instruments Inc., PA, USA).

#### 2.3.4. Sucrose Preference Test

The sucrose preference test is used as an indicator of anhedonic behavior in rodents. Before the beginning of testing, rats were kept individually habituated to two needleless syringes (filled with 1% sucrose solution and plain water, resp.) 8 h per day for 2 days in their home cage. After training, the rats were deprived of food and water for 12 h and then exposed to the two syringes for 30 min as described previously [32]. After an interval of 1 h, the positions of the syringes were exchanged, and the rats were tested for 30 min again. The volume of water and sucrose solution intake during 1 h was measured. Sucrose preference was calculated as a percentage of the volume of sucrose intake to the total volume of fluid intake.

#### 2.3.5. Forced Swim Test

The forced swim test (FST) is commonly used to assess behavioral despair in rodents [22, 23]. The protocol consists of a 15-min pretest swim and a 5-min test swim on the following day. Rats were placed into a cylindrical receptacle (height, 60 cm; diameter, 26 cm) filled with water (25°C, 35 cm deep). Behavioral activity was recorded for 5 min using a digital video camera connected to SMART video-tracking system (Panlab, Spain). The amount of time spent in a posture of immobility (i.e., the lack of motion of the entire body, with the exception of only small movements necessary to keep the animal's head above the water), swimming (i.e., large forepaw movements that displaced water and moved the animal's body around the cylinder, which were more than necessary to keep the head above the water), and climbing (i.e., vigorous movements of the forepaws in and out of the water, usually directed against the wall of the tank) was calculated. All the behavioral tests were performed following randomization and blinding.

### 2.4. TH Immunohistochemical Analysis and Quantification

Rats were sacrificed and transcardially perfused with 4% paraformaldehyde in 0.1 M phosphate buffer. Frozen mesencephalon coronal sections were cut into 30-*μ*m thick slices on a cryostat and processed for TH immunohistochemical analysis. Brain slices were incubated overnight at 4°C with TH antibody (diluted 1 : 2000, Chemicon, USA), followed by incubation with a biotinylated secondary antibody (Lab Vision, USA) for 30 min. The antibody was detected with an avidine/biotine complex (Vector Laboratories, USA) and visualized with 3,3-diaminobenzidine (Sigma). Six identical TH-labeled slices spanning the entire mesencephalon and including both SNc and VTA brain regions were selected from 5 animals per group. Using a bright-field microscope (Olympic, Japan), the boundaries of the SNc and VTA areas were drawn. The number of TH-immunoreactive profiles in these areas was counted by stereology, using Stereo Investigator software (MBF Bioscience, USA) as described previously [33]. The survival percentage of TH-positive cells was calculated as a percentage of the number of surviving cells on the lesioned side to that on the nonlesioned side. An independent investigator evaluated all sections in a blinded manner.

### 2.5. Quantification of DA, 5-TH, and NE

Rats were sacrificed by decapitation 1 day after behavioral measurements. The striatum, ventral midbrain, and hippocampus were rapidly dissected and stored in liquid nitrogen at –80°C. The concentrations of DA, 5-HT, and NE were assayed using reverse-phase high-performance liquid chromatography (HPLC) with electrochemical detection. The tissue samples were homogenized with an ultrasonic cell disrupter (Sonics) in 0.4 M perchloric acid. After centrifugation at 10,000 ×g for 30 min, the supernatant (160 *μ*L) was mixed with 80 *μ*L mobile phase solution and was kept in the dark in an ice-bath (60 min), followed by centrifugation (12,000 rpm, 20 min). The resultant supernatant was injected into the chromatograph. The mobile phase solution (flow rate, 1 mL/min) comprised 63.5 mM citric acid monohydrate, 60.9 mM trisodium citrate dihydrate, 0.1 mM EDTA, 0.5 mM 1-octanesulfonic acid sodium salt, and 8% methanol. The pH of the running buffer solution was adjusted to 4.0, and it was then filtered through a filter of pore size 0.45 mm. The peak areas of external standards were used to quantify sample peaks.

### 2.6. Enzyme-Linked Immunosorbent Assay

The level of BDNF was determined by enzyme-linked immunosorbent assay (ELISA). The ventral midbrain and hippocampus were rapidly dissected and homogenized in a lysis buffer containing protease inhibitors (Sigma-Aldrich). The sample protein contents were measured using a BCA protein assay kit (Pierce, Rockford, IL). For ELISA, a BDNF ELISA kit was used according to the manufacturer's instructions (Millipore, Billerica, MA). BDNF levels were determined relative to a standard curve constructed using BDNF protein standards that were assayed simultaneously with the experimental samples.

### 2.7. Western Blotting

The expression of TrkB was determined by western blotting. The ventral midbrain and hippocampus tissues of rats were prepared as described previously [32]. Equivalent amounts of proteins were processed for SDS-PAGE and electrotransferred onto Immobilon-P membranes (Millipore, Billerica, MA). After they were blocked with 5% nonfat milk in 0.5% Tween 20 in 20 mM Tris and 137 mM NaCl for 1 h at room temperature, the membranes were incubated overnight at 4°C with anti-TrkB (1 : 3000, Cell Signaling). The membranes were probed with a mouse anti-actin antibody as an internal control. The blots were then incubated for 1 h at room temperature with horseradish peroxidase-conjugated secondary antibody. Protein bands were detected using the ECL. Western Blotting Substrates (Pierce, Rockford, IL) were estimated using the Image Analysis Program Labwork 4.5.

### 2.8. Statistical Analysis

Data were reported as means ± SEM. One- or two-way analysis of variance (ANOVA) was used, as appropriate, followed by the Newman-Keuls* post hoc* test for comparison of groups. *P* < 0.05 was the criterion for statistical significance.

## 3. Results

### 3.1. Effects of EA on Motor Function and Dopaminergic Neurodegeneration in 6-OHDA Lesioned Rats

**Figure 1:**
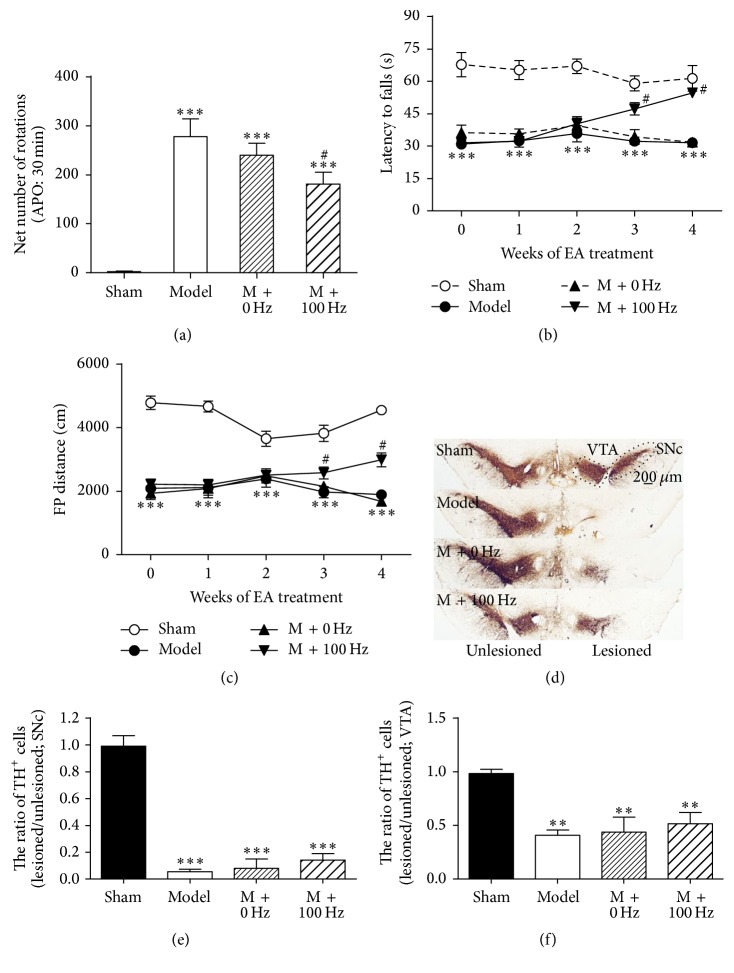
Effects of 100 Hz EA treatment on motor symptoms and dopaminergic neurodegeneration in the ventral midbrain of 6-OHDA lesioned rats. ((a)–(c)) Effects of EA on APO-induced rotational behavior (a), rotarod test (b), and horizontal movement distance in the open field test (c). Data represent mean ± SEM (*n* = 9–11 per group). ^*∗∗*^ *P* < 0.01 and ^*∗∗∗*^ *P* < 0.001 versus the sham group; ^#^ *P* < 0.05 versus the model group. (d) Effects of EA on TH immunohistochemical staining. SNc is implied by the elliptical dotted line and VTA is implied by rectangle dotted line in (d). ((e) and (f)) Quantification of the damaged extent of TH-positive profiles on lesioned compared to unlesioned side in the SNc (e) and in the VTA (f). Data represent mean ± SEM, ^*∗∗*^ *P* < 0.01, and ^*∗∗∗*^ *P* < 0.001 versus the sham group.

The apomorphine-induced turning test was performed 6 weeks after 6-OHDA lesions were induced in the rats. As shown in Figure 1(a), unilateral injection of 6-OHDA into the MFB produced intense contralateral turning behavior compared to the sham group (*P* < 0.05), while 100-Hz EA treatment, but not 0-Hz EA treatment, reduced the intensity of apomorphine-induced turning. Sham animals (*n* = 19) showed no turning behavior. Motor coordination was assessed using the rotarod test (Figure 1(b)). A repeated two-way ANOVA showed an effect of group (*F*_3,34_ = 71.55; *P* < 0.001) and weeks of treatment (*F*_4,34_ = 1.39; *P* = 0.25) and an interaction of group × weeks (*F*_12,34_ = 4.53, *P* < 0.001). Further* post hoc* analyses showed that 6-OHDA-lesioned rats developed significant coordination deficits (*F*_3,34_ = 24.35, *P* < 0.001) before EA treatment. However, 100-Hz EA did not reverse this latency after 1 (*P* > 0.05) or 2 weeks of treatment (*P* > 0.05) but showed some effect following 3 (*P* < 0.001) and 4 weeks (*P* < 0.001) of treatment compared with the 6-OHDA model group. Meanwhile, an obvious decrease in the horizontal movement distances was found in the 6-OHDA group compared to the sham group (*F*_3,30_ = 34.86, *P* < 0.001) before EA treatment. However, 100-Hz EA significantly increased horizontal movement distances after 3 (*P* < 0.001) and 4 weeks (*P* < 0.001) of treatment. Similar to previous reports, 0-Hz EA showed no effect on these motor deficits [11]. These data suggest that long-term 100-Hz EA stimulation might improve motor function in 6-OHDA model rats.

Immunohistochemical staining for TH was performed 6 weeks after the MFB lesions were induced, and the numbers of TH-immunoreactive neurons in the SNc and VTA were quantified. The data show that 6-OHDA MFB lesions induced loss of TH-positive profiles by 94.5% ± 1.8% in the SNc on the ipsilateral side compared with the unlesioned side (*P* < 0.001, Figures 1(d) and 1(e)). 100-Hz EA stimulation for 4 weeks showed no effects on the degeneration of TH-positive cells in the SNc compared with the 6-OHDA group (*P* > 0.05, Figures 1(d) and 1(e)). Noticeably, the TH-positive profiles decreased by 40.81% ± 4.91% in the VTA of the lesioned side compared with the contralateral side (Figures 1(d) and 1(f)). However, an increase in TH-positive profiles in the ipsilateral VTA was observed after 4 weeks of 100-Hz EA (Figure 1(f)), although this change was not significant (*P* > 0.05). These results collectively support the contention that 100-Hz EA can alleviate the abnormal movement symptoms triggered by 6-OHDA lesion and has a minimal impact on the mesocorticolimbic dopaminergic pathway but not on the mesostriatal pathway.

### 3.2. Effects of EA on Depressive-Like Symptoms in 6-OHDA-Lesioned Rats

**Figure 2:**
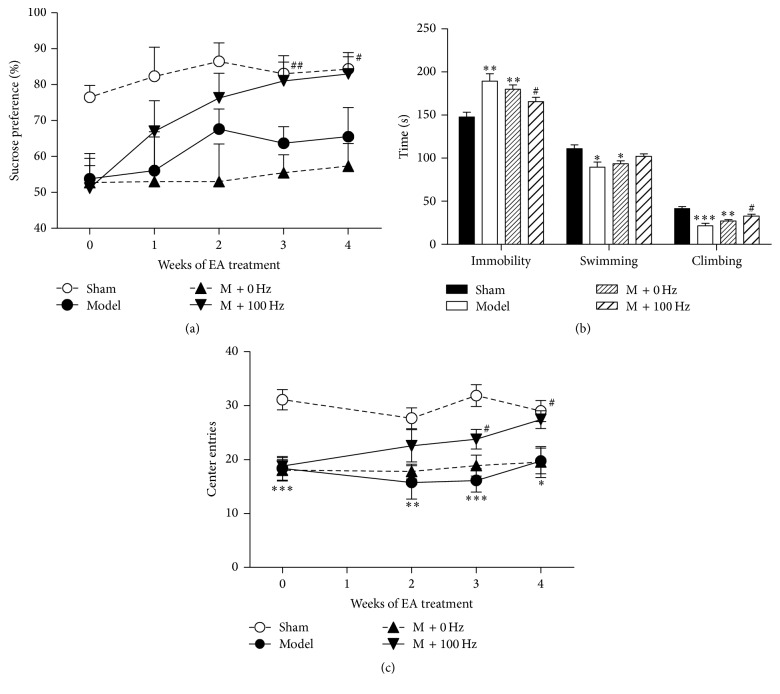
EA at 100 Hz improved depressive-like symptoms in 6-OHDA lesioned rats. (a) Changes of sucrose intake in sucrose preference test. (b) Changes of the immobility and swimming and climbing behaviors in the forced swim test. (c) Changes of the center entries in the open field test. Data represent mean ± SEM (*n* = 9–11 per group). ^*∗*^ *P* < 0.05, ^*∗∗*^ *P* < 0.01, and ^*∗∗∗*^ *P* < 0.001 versus the sham group; ^#^ *P* < 0.05 and ^##^ *P* < 0.01 versus the model group.

Depressive-like behavioral phenotypes were assessed after 4 weeks of EA treatment. Anhedonic behavior was measured using the sucrose preference test. Two-way ANOVA showed significant effects of group (*F*_3,180_ = 14.62, *P* < 0.0001) and week (*F*_4,180_ = 2.62, *P* = 0.0365) but no significant effect of the group × week interaction (*F*_12,180_ = 0.567, *P* = 0.867). Bonferroni* post hoc* analysis shows that all 6-OHDA-lesioned rats displayed a significant decrease in sucrose solution intake 2 weeks after neurotoxin exposure compared to the sham rats (*P* < 0.05; Figure 2(a)). The 100-Hz group showed a weekly increase in sucrose preference and a significant increase after 3 (*P* < 0.05) and 4 weeks (*P* < 0.05) of EA treatment compared with the 6-OHDA group.

FSTs were performed to evaluate behavioral despair in depressive rats. Six weeks after neurotoxin exposure, the 6-OHDA group exhibited a significant increase in immobility time compared with the sham group (*P* < 0.05). However, 4 weeks of EA treatment effectively reduced immobility time compared to 6-OHDA group (*P* < 0.05). Analysis of swimming and climbing behavior in the FST demonstrated that 6-OHDA MFB lesions significantly affected these parameters (all *P* < 0.05). Similarly, EA treatment effectively increased climbing time compared to 6-OHDA group (*P* < 0.05).

Moreover, the open field test was used to assess anxiety-related behavior. Two-way ANOVA showed significant effects of group (*F*_3,124_ = 24.4, *P* < 0.001) but no significant effects of week (*F*_3,124_ = 1.49, *P* = 0.221) and interaction between group and week (*F*_9,124_ = 0.988, *P* = 0.453) (Figure 2(c)). In the open field test, 6-OHDA-lesioned rats showed fewer central square entries (*P* < 0.001, Figure 2(c)) than sham rats. This result suggests that 6-OHDA induces an increase in anxiety-related behavior. However, the 100-Hz EA group displayed an increase in the number of entries after 3 (*P* < 0.05) and 4 weeks (*P* < 0.05) of treatment. Thus, continuous EA stimulation has a strong positive effect on reversing the anhedonic-like and despair-like behavioral phenotypes in 6-OHDA-lesioned rats.

### 3.3. Effects of EA on the DA, 5-HT, and NE Levels in the Striatum and Hippocampus

**Figure 3:**
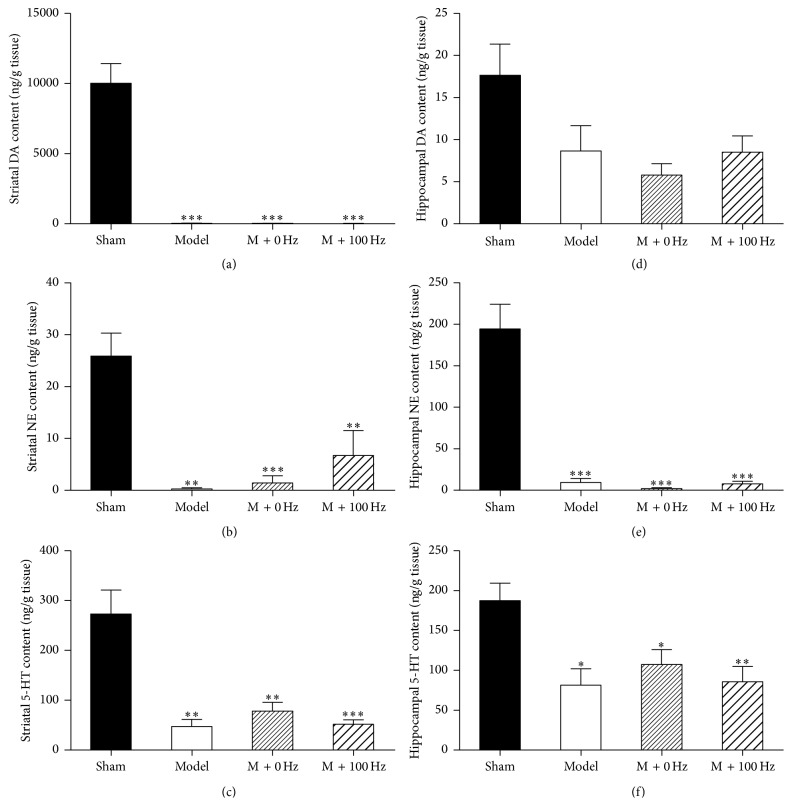
EA at 100 Hz had no significant effects on the contents of DA, NE, and 5-HT in the striatum ((a)–(c)) and that in the hippocampus ((d)–(f)). The content of transmitters was measured by HPLC. Data represent mean ± SEM (*n* = 5-6 per group). ^*∗*^ *P* < 0.05, ^*∗∗*^ *P* < 0.01, and ^*∗∗∗*^ *P* < 0.001 versus the sham group.

To determine whether EA exerts its therapeutic effects by regulating neurotransmitters, the levels of DA, 5-HT, and NE in the striatum and hippocampus were measured after 4 weeks of 100-Hz EA treatment. As shown in Figure 3(a), the level of DA was almost completely depleted in the 6-OHDA-lesioned ipsilateral striatum compared with that in the sham group (*P* < 0.001). Similarly, the DA has a tendencious but not significant decrease in the lesioned ipsilateral hippocampus compared with the level in the sham group (*P* = 0.095, Figure 3(d)). Neither level 100-Hz nor level 0-Hz EA produced any effect on the DA concentration either in the striatum (Figure 3(a)) or in the hippocampus (Figure 3(d)). The 5-HT and NE levels were also remarkably reduced in the lesioned hemisphere compared to the unlesioned hemisphere, both in the striatum (*P* < 0.001) and in the hippocampus (*P* < 0.001). Additionally, the 5-HT and NE levels in the striatum were unaffected by 4 weeks of treatment with 100-Hz EA (Figures 3(a), 3(b), and 3(c)), and similar results were observed in the hippocampus (Figures 3(d), 3(e), and 3(f)). These findings suggest that 100-Hz EA has no influence on the levels of DA, 5-HT, and NE in the striatum and hippocampus.

### 3.4. Effects of EA Treatment on the Expression of BDNF and TrkB in the Hippocampus and Ventral Midbrain

**Figure 4:**
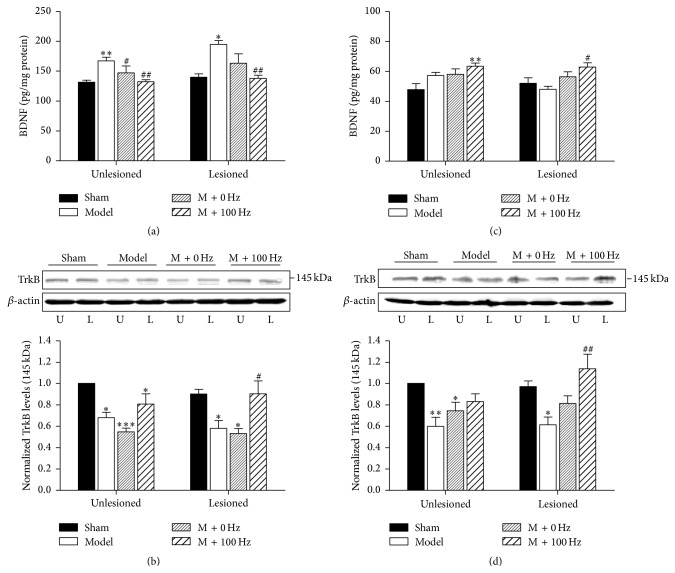
EA at 100 Hz modulated the abnormal expression of BDNF and TrkB in the midbrain and in the hippocampus. BDNF levels were measured by ELISA on the lesioned side and unlesioned side in the ventral midbrain (a) and the hippocampus (c), respectively. TrkB expressions were measured by western blotting on the unlesioned side and lesioned side in the ventral midbrain (b) and the hippocampus (d), respectively. *β*-actin was used as an internal control. Data represent mean ± SEM (*n* = 5-6 per group). ^*∗*^ *P* < 0.05, ^*∗∗*^ *P* < 0.01, and ^*∗∗∗*^ *P* < 0.001 versus the sham group; ^#^ *P* < 0.05 and ^##^ *P* < 0.01 versus the model.

To explore the possible mechanisms involved in the antidepressive effects of 100-Hz EA, the rats were sacrificed after 4 weeks of EA treatment. As shown in Figures 4(a) and 4(c), 6-OHDA lesions induced an increased BDNF level (*P* < 0.05) on the lesioned side of ventral midbrain and a decreased BDNF level (*P* < 0.05) on the lesioned side of hippocampus compared with the sham group, respectively. However, these alterations in BDNF levels on the lesioned side induced by 6-OHDA were reversed by EA treatment both in the ventral midbrain and in the hippocampus. Although the changes in BDNF level on the unlesioned side were modulated by EA treatment in the ventral midbrain, it has no influence on the unlesioned side in the hippocampus (Figures 4(a) and 4(c)). Meanwhile, there were significant decreases of TrkB expression on the lesioned side both in the ventral midbrain and in the hippocampus compared with the sham group (*P* < 0.01). 100 Hz EA treatment could increase TrkB expression on the lesioned side both in the ventral midbrain and in the hippocampus (Figures 4(b) and 4(d)). Similarly, EA treatments have no obvious effects on the TrkB expression on the unlesioned side in the ventral midbrain or the hippocampus. These data suggest that 100-Hz EA treatment could modulate 6-OHDA-induced abnormal expression of BDNF and TrkB on the lesioned side in the ventral midbrain and hippocampus.

## 4. Discussion

### 4.1. EA Improves Depressive Symptoms and Partly Affects VTA Dopaminergic Pathways

The core biochemical dysfunction that primarily, but not exclusively, contributes to the motor features in PD is the profound deficit in the DA level. When classic motor deficits (including resting tremor, rigidity, and bradykinesia) occur, there are more than 50% of dopaminergic neurons that are lost in the SNc, exceeding 60–80% DA depletion in the striatum [34–37]. In the unilateral PD model used in the present study, almost all the dopaminergic neurons were lost in the SNc and the DA level was depleted 7-fold in the ipsilateral striatum after 6-OHDA lesions were induced in the MFB. The results of the apomorphine-induced rotation, open field, and rotarod tests strongly suggest that the motor hallmarks of PD are closely associated with mesostriatal dopaminergic degeneration and basal ganglia circuit dysfunction [25, 29].

Previous studies indicated that the 6-OHDA MFB lesion model is a relevant tool to study the pathophysiology of both the motor and nonmotor symptoms of PD, including severe motor dysfunction and depressive-like symptoms [29]. These behavioral alterations can be attributed to the significant disruption in both the mesostriatal and mesocorticolimbic dopaminergic pathways. In the present experiment, the depressive symptoms of PD were measured using the sucrose preference test, which has been widely used to assess the anhedonic response in animal models of depression [38]. Two weeks after 6-OHDA administration, a significant decrease in sucrose consumption was found in the 6-OHDA-lesioned animals. The FST is a well-established behavioral assessment tool used to evaluate depressive-like features; particularly the prolonged immobility can be regarded as “behavioral despair.” Our data showed that the 6-OHDA-lesioned model showed a high immobility time. Additionally, a significant reduction in dopaminergic neurons in the VTA and a tendencious but not significant decrease of the DA level in the hippocampus were found in the 6-OHDA-lesioned rats. Recent data suggest that damage to the dopaminergic neurons in the VTA affecting the ascending mesolimbic and mesocortical pathways leads to a depressive phenotype in rats and remarkable low swimming and climbing times. Meanwhile, the number of central square entries in the open field test was markedly reduced in the lesioned rats. Consistent with prior data [29], our results supported the contention that rats with complete unilateral dopaminergic neuron damage, that is, depletion of both mesocorticolimbic and nigrostriatal DA, develop severe motor impairments and depressive-like symptoms [28]. Clinical evidence has also shown that dopamine dysfunction in PD-associated depression is mainly related to DA projections arising from neurons in the VTA [39]. In support of these data, previous neurochemical evidence has shown that mesencephalic dopaminergic projection to the hippocampus declined after 6-OHDA lesions in the SNc [40]. This situation mimics a mild damage of PD where mesencephalon-limbic dopaminergic pathways are affected [41]. Accordingly, HPLC analysis in the present study showed that the hippocampal content of DA in the 6-OHDA-lesioned animals was significantly reduced.

In the present study, we demonstrated that high-frequency EA improves the motor symptoms as well as the nonmotor symptoms in PD rats. Similar to our previous studies [11, 15], this study also showed that 100-Hz EA alleviated abnormal movement symptoms but had no effect on nigrostriatal dopaminergic transmission. Interestingly, we found that EA produced a minimal neuroprotective impact on VTA dopaminergic neurons, although this result was not statistically significant. Thus, EA seems to affect mesencephalolimbic dopaminergic pathways and improve depressive symptoms.

### 4.2. EA Modulates BDNF-TrkB Signals but Has No Effect on the Noradrenergic and Serotonergic Systems

Although PD is widely known to be a disorder associated with the depletion of striatal DA, prior evidence has indicated that dysfunctions of other neurotransmitters, such as NE and 5-HT, also participate in the development of depression in PD [42]. According to the Braak staging of PD pathology, NA and 5-HT dysfunction occur prior to the significant degradation of dopaminergic neurons [4].

Clinical evidence has also shown that 5-HT alterations occur in patients with PD-associated depression [36]. The administration of 6-OHDA in the MFB resulted in noradrenergic and serotonergic reduction in the striatum and hippocampus. On the basis of the known role of the serotonergic system in emotional processes, it is conceivable that reduced 5-HT levels are closely related to mood-related phenotypes [24]. The 6-OHDA-induced PD model produced sustained depressive-like behaviors, including anhedonia and behavioral despair, which are directly associated with the reduction in striatal DA and hippocampal 5-HT content [26]. In addition, decreased NE levels in the hippocampus and significant losses in limbic noradrenergic structures have also been found in PD patients [37]. Neuroimaging studies have shown that significant losses in limbic noradrenergic structures might be associated with depression in PD [31].

In the current study, we found that 6-OHDA induced a marked reduction in the levels of DA, 5-HT, and NE in both the striatum and hippocampus, which suggests that dysfunctions in these neurotransmitters may contribute to many nonmotor symptoms of PD. Thus, correlations may exist between swimming in the FST and the serotonergic system or between immobility in the FST and the dopaminergic system. Therefore, one or more of these neurotransmitter systems may play an important role in depressive-like behavior in this PD model, and interventions aimed at restoring monoamine function may be beneficial in treating the disease [24, 36]. However, neither 100-Hz nor 0-Hz EA elicited any significant changes in the content of DA, 5-HT, or NE either in the striatum or in the hippocampus in the 6-OHDA-lesioned rats. Therefore, the mechanism of action of EA treatment does not seem to be related to these neurotransmitters.

As a key regulator of synaptic development and plasticity, BDNF has been recognized to play a pivotal role in several neurodegenerative and psychiatric disorders [24]. Our previous experiment suggested that high-frequency EA plays a neurotrophic role in PD rats by increasing BDNF mRNA levels in the SNc and VTA [15]. The protective properties of acupuncture might be mediated by TrkB receptors in the 6-OHDA model [43]. Thus, our study revealed the regulatory effect of EA on BDNF-TrkB signaling.

Correlations between depressive-like symptoms and the reduction in BDNF mRNA and protein levels in the hippocampus have been identified in postmortem evidence [16, 17]. Further, enhanced BDNF expression has been found in the nucleus accumbens of individuals with depression [44]. These alterations were reversed following antidepressant treatment [45, 46]. In experimental models, BDNF levels decreased in the hippocampus and frontal cortex [20, 21], whereas they increased in the nucleus accumbens and VTA [22, 47], which might suggest different roles of BDNF in different brain structures [27]. Similarly, we also observed a decrease in BDNF levels and TrkB expression in the hippocampus after 6-OHDA injection. Notably, 4 weeks of EA treatment remarkably increased the BDNF levels and TrkB expression in the hippocampus. Thus, the antidepressant role of EA may activate a cascade of neurotrophic activity including BDNF and TrkB within the mesocorticolimbic dopaminergic system, which originates from the ventral tegmental area and innervates the thalamus and hippocampus.

In contrast, 6-OHDA lesions induced an increase in BDNF expression in the midbrain. There are some complex reasons as to why this may be. Because BDNF is highly expressed by dopaminergic neurons, the degeneration of dopaminergic neurons should induce decreased BDNF expression. However, it has been previously shown that 6-OHDA injection triggers microglia and astrocyte proliferation and increases BDNF-immunoreactivity in the lesioned midbrain [48, 49]. In addition, the SNc and VTA can project to the hippocampus through the activation of 5-HT_2A_ receptors to alter serotonin transmission [50]. Therefore, there should be a correlation between the reduced BDNF expression in the hippocampus and increased BDNF expression in the midbrain. Moreover, decreased expression of TrkB in the midbrain was also observed in the 6-OHDA model [43]. This discrepancy between the changes in BDNF and TrkB levels has not been clarified thus far and requires further investigation.

In the present study, alterations in the BDNF levels and TrkB expression induced by 6-OHDA lesions were reversed by EA treatment in the hippocampus and midbrain. Therefore, we concluded that EA might modulate BDNF-TrkB interaction in the mesolimbic dopaminergic pathway. Moreover, BDNF seems to participate in the effects of EA and may serve as a potential therapeutic target in PD.

## 5. Conclusion

In summary, we demonstrated that high-frequency EA improves the motor symptoms as well as the nonmotor symptoms in PD rats. The effects of EA in improving depressive-like symptoms might be partially attributed to the neuroprotective role of EA in the mesocorticolimbic dopaminergic pathways. 100-Hz EA did not elicit any significant changes in the level of 5-HT and NE either in the striatum or in the hippocampus of the 6-OHDA-lesioned rats. Interestingly, BDNF-TrkB pathway seems to participate in the effect of EA and may serve as potential therapeutic targets in PD.
